# Impact of Supercritical Carbon Dioxide on Pore Structure and Gas Transport in Bituminous Coal: An Integrated Experiment and Simulation

**DOI:** 10.3390/molecules30061200

**Published:** 2025-03-07

**Authors:** Kui Dong, Zhiyu Niu, Shaoqi Kong, Bingyi Jia

**Affiliations:** 1 College of Geological and Surveying Engineering, Taiyuan University of Technology, Taiyuan 030024, China; dongkui@tyut.edu.cn (K.D.); 15525037025@163.com (Z.N.); 2 College of Mining Engineering, Taiyuan University of Technology, Taiyuan 030024, China; 3 School of Safety Science and Engineering, Xi’an University of Science and Technology, Xi’an 710000, China; jiabingyiccteg@126.com; 4 Xi’an Research Institute of China Coal Technology and Engineering Group Corp., Xi’an 710000, China

**Keywords:** ScCO_2_, bituminous, pore structure, molecular structure, diffusion

## Abstract

The injection of CO_2_ into coal reservoirs occurs in its supercritical state (ScCO_2_), which significantly alters the pore structure and chemical composition of coal, thereby influencing the adsorption and diffusion behavior of methane (CH_4_). Understanding these changes is crucial for optimizing CH_4_ extraction and improving CO_2_ sequestration efficiency. This study aims to investigate the effects of ScCO_2_ on the pore structure, chemical bonds, and CH_4_ diffusion mechanisms in bituminous coal to provide insights into coal reservoir stimulation and CO_2_ storage. By utilizing high-pressure CO_2_ injection adsorption, low-pressure CO_2_ gas adsorption (LP-CO_2_-GA), Fourier-transform infrared spectroscopy (FTIR), and reactive force field molecular dynamics (ReaxFF-MD) simulations, this study examines the multi-scale changes in coal at the nano- and molecular levels. The following results were found: Pore Structure Evolution: After ScCO_2_ treatment, micropore volume increased by 19.1%, and specific surface area increased by 11.2%, while mesopore volume and specific surface area increased by 14.4% and 5.7%, respectively. Chemical Composition Changes: The content of aromatic structures, oxygen-containing functional groups, and hydroxyl groups decreased, while aliphatic structures increased. Specific molecular changes included an increase in (CH_2_)_n_, 2H, 1H, and secondary alcohol (-C-OH) and phenol (-C-O) groups, while Car-Car and Car-H bonds decreased. Mechanisms of Pore Volume Changes: The pore structure evolves through three distinct phases: Swelling Phase: Breakage of low-energy bonds generates new micropores. Aromatic structure expansion reduces intramolecular spacing but increases intermolecular spacing, causing a decrease in micropore volume and an increase in mesopore volume. Early Dissolution Phase: Continued bond breakage increases micropore volume, while released aliphatic and aromatic structures partially occupy these pores, converting some mesopores into micropores. Later Dissolution Phase: Minimal chemical bond alterations occur, but weakened π-π interactions and van der Waals forces between aromatic layers result in further mesopore volume expansion. Impact on CH_4_ Diffusion: Changes in pore volume directly affect CH_4_ migration. In the early stages of ScCO_2_ interaction, pore shrinkage reduces the mean square displacement (MSD) and self-diffusion coefficient of CH_4_. However, as the reaction progresses, pore expansion enhances CH_4_ diffusion, ultimately improving gas extraction efficiency. This study provides a fundamental understanding of how ScCO_2_ modifies coal structure and CH_4_ transport properties, offering theoretical guidance for enhanced CH_4_ recovery and CO_2_ sequestration strategies.

## 1. Introduction

The deep CO_2_ sequestration technology for enhancing coalbed methane recovery (CO_2_-ECBM) not only sequesters CO_2_ but also increases the productivity of coalbed methane, making it a significant direction for future industrial development [1,2,3]. The treatment of coal with supercritical fluids, including CO_2_, has been widely explored for various purposes, such as desulfurization, the extraction of specific compounds, and the modification of coal properties to enhance methane recovery [4]. Supercritical fluids possess unique properties, including high diffusivity, low viscosity, and tunable solvating ability, allowing them to penetrate coal structures efficiently and facilitate physical and chemical interactions. Further, the purpose of coalbed methane recovery should be described. The primary objective of coalbed methane (CBM) recovery is to extract methane gas from coal seams efficiently while minimizing environmental impacts. CBM is an important unconventional natural gas resource, and its extraction contributes to energy security, reduces greenhouse gas emissions by capturing methane that would otherwise be released into the atmosphere, and provides a cleaner-burning alternative to traditional fossil fuels.

The treatment of coal with supercritical fluids, particularly ScCO_2_, aims to enhance coalbed methane extraction and improve CO_2_ sequestration efficiency. Supercritical fluids exhibit unique solvent properties, such as high diffusivity, low viscosity, and the ability to penetrate coal matrices more effectively than conventional gases or liquids. These characteristics allow ScCO_2_ to dissolve and alter organic components within the coal, influencing porosity, permeability, and gas transport dynamics. The objective of ScCO_2_ treatment is to induce structural modifications in coal that enhance its methane release capacity while simultaneously increasing CO_2_ adsorption sites, thus improving the feasibility of long-term CO_2_ storage in coal seams.

As practical applications of CO_2_-ECBM technology advance, associated challenges have come to the forefront. The CO_2_ injected into coal reservoirs exists as a supercritical fluid (ScCO_2_) [5,6,7], and its unique properties facilitate complex physical and chemical reactions with the coal seams. This interaction can alter the pore structure and chemical composition of the coal, significantly influencing its gas adsorption, diffusion, and transport capacities [8,9], thereby affecting the efficiency of CO_2_ in displacing CH_4_ and the safety of CO_2_ sequestration. Consequently, understanding the impact of ScCO_2_ on coal seams and the underlying mechanisms has become a focus of research. Due to bituminous coal’s high volatile matter content, abundance of functional groups, porous structure, diversity, and high methane production potential, studying the interaction between CO_2_ and bituminous coal is crucial [10]. This understanding is fundamental for elucidating the dynamic evolution characteristics of coal reservoirs during coalbed methane production, as well as the behavior and mechanisms of fluid interactions in CO_2_-ECBM technology.

Some researchers have studied the changes in the pore structure of bituminous coal after exposure to ScCO_2_. Wang et al. thought that after exposure to ScCO_2_, the volumes of micropores, mesopores, and macropores in bituminous coal all increase [11]. According to Guan et al., as the reaction time between ScCO_2_ and bituminous coal increases, the micropore volume first increases, then decreases, and increases again; the mesopore volume first increases and then decreases; and the total pore volume shows a trend of increasing, decreasing, and then increasing [12]. Liu et al. found that after exposure to ScCO_2_, the total pore volume in bituminous coal increases, while the average pore diameter decreases, indicating an increase in micropore content [13]. Su et al. observed that after exposure to ScCO_2_, the micropore content in bituminous coal decreased, while the macropore content increased [14]. Zhang et al. suggested that after exposure to ScCO_2_, the micropores in bituminous coal transform into mesopores, resulting in a decrease in micropores and an increase in mesopores [15].

The pore structure of coal significantly affects gas diffusion behavior. Wu et al., using molecular dynamics simulations, investigated the adsorption and diffusion behaviors of CO_2_ and CH_4_ in shale. They found that increasing particle size at a given porosity facilitates faster adsorption/desorption of CO_2_ and CH_4_ within the solid matrix and enhances the diffusion rates [16]. Liu et al. employed Fick’s law to study gas diffusion after CO_2_ injection [17]. They reported that due to swelling and acid dissolution effects, the number of fractures in coal increases, resulting in higher diffusion coefficients and adsorption capacities for both CO_2_ and CH_4_. Other researchers have obtained similar conclusions, further supporting the significant impact of pore structure on gas diffusion in coal [18,19].

Approximately 90% of methane in coal is stored in micropores (<10^−9^ m) and mesopores (10^−9^–10^−8^ m), which are crucial for the adsorption and diffusion of coalbed methane [20,21,22]. Additionally, changes in the chemical structure can further influence the pore structure. Based on the above considerations, this study focuses on investigating the effects of supercritical CO_2_ (ScCO_2_) on the pore structure and chemical composition of bituminous coal. The primary aim is to elucidate the dynamic evolution of coal reservoirs during CO_2_-enhanced coalbed methane recovery (CO_2_-ECBM) and to provide a theoretical basis for optimizing methane extraction and CO_2_ sequestration in bituminous coal seams. To achieve this, Tunlan (TL) bituminous coal is selected as the research subject. The study is carried out through a combination of experimental and simulation approaches. Experimentally, high-pressure CO_2_ adsorption, low-pressure CO_2_ gas adsorption (LP-CO_2_-GA), and Fourier-transform infrared spectroscopy (FTIR) are conducted to analyze changes in the pore structure and chemical bonds after exposure to ScCO_2_. In parallel, ReaxFF molecular dynamics (ReaxFF-MD) simulations are utilized to explore the mechanisms underlying these changes at the nano- and molecular scales. The specific tasks of this research include (1) characterizing the evolution of coal’s pore structure during different exposure periods to ScCO_2_; (2) analyzing the variations in chemical bonds and functional groups induced by ScCO_2_ interactions; (3) using reactive force field molecular dynamics (ReaxFF-MD) simulations to explore the dynamic processes governing pore structure modification and chemical reactions at the molecular scale; and (4) investigating the diffusion behaviors of CO_2_ and CH_4_ in the modified coal matrix. The findings aim to deepen the understanding of ScCO_2_–coal interactions, ultimately supporting the safe and efficient implementation of CO_2_-ECBM technology.

## 2. Samples and Methodology

**Figure 1:**
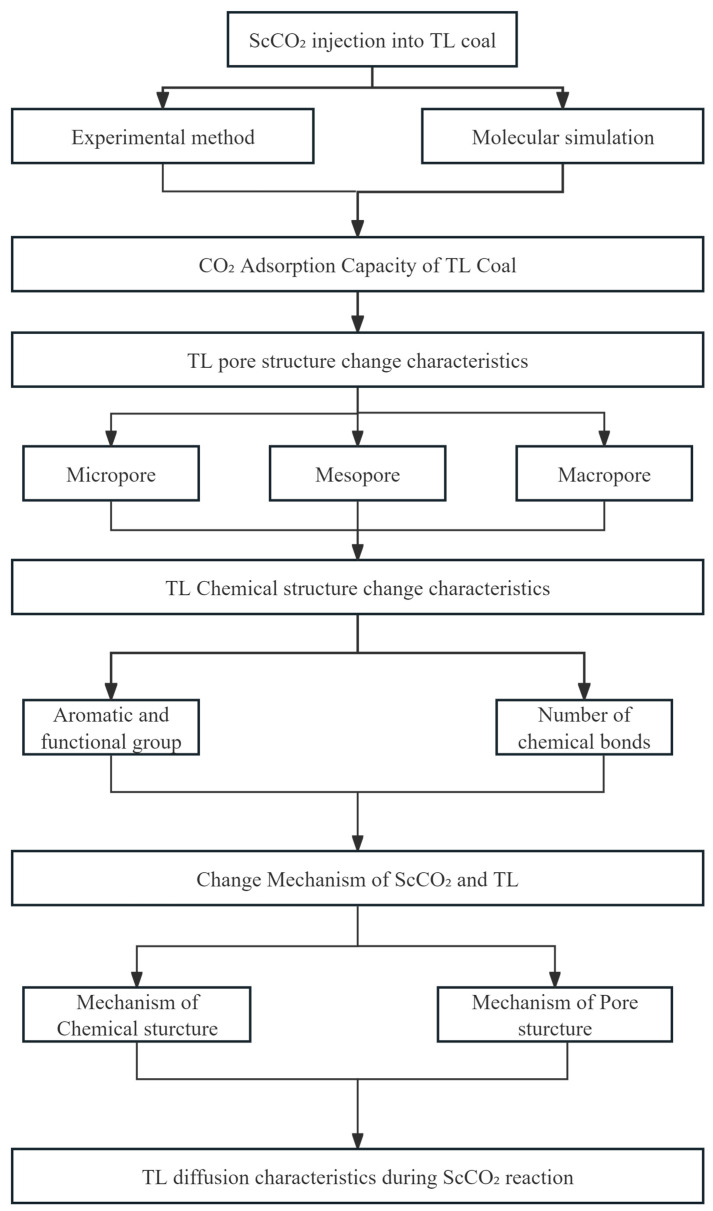
ScCO_2_ and TL interaction mechanism analysis flow chart.

In this study, a series of experimental and computational methods were employed to investigate the effects of supercritical ScCO_2_ on the pore structure and chemical composition of bituminous coal. The flow chart is shown in Figure 1.

### 2.1. Sample

The coal samples were collected from the Tunlan Mine (TL) in the Xishan Coalfield, Shanxi. After assessing the geological conditions of the mine and identifying the sampling points, block samples were collected using a pickaxe in a newly excavated coal seam. The sampling process was carried out following the standard procedure outlined in Methods for the Sampling of Coal and Coke (GB/T 19222-2003, 2003) [23]. The collected coal samples were wrapped with wide tape, sealed with wax, and then packed for transportation. The proximate analysis results were obtained according to Proximate Analysis of Coal (GB/T 212-2008, 2008) [24].

The coal samples were crushed to a particle size of 60–80 mesh for vitrinite reflectance determination and FTIR experiments and to below 200 mesh for proximate analysis and LP-CO_2_-GA experiments. An elemental analysis of sample TL was conducted using a Vario EL elemental analyzer from Elementar Analysen systeme GmbH, Langenselbold, Germany. The proximate and elemental analysis data of the samples are shown in Table 1. The coal samples used in this study were collected from the TL coal seam in the Ximing Mine, Shanxi Province. According to proximate and ultimate analysis (Table 1), the coal sample has a volatile matter content of 23.9% and a fixed carbon content of 86.34%. Based on the classification criteria in the classification of coals (ISO 11760:2005) [25], the measured volatile matter and calorific value confirm that the sample belongs to the bituminous coal category.

### 2.2. ScCO_2_ Treatment

The temperature of the isothermal box was set to 50 °C, ensuring that the experiment mimicked deep-coal-seam conditions. ScCO_2_ (purity: 99.99%) was injected into the reference vessel. Once the system’s temperature and pressure stabilized, the valve was opened to transfer CO_2_ from the reference vessel to the sample vessel. The valve was closed when the gas pressure in the sample vessel reached 10 MPa, a pressure well above the critical pressure of CO_2_ (32 °C, 7.38 MPa), to maintain its supercritical state. An adsorption equilibrium was considered to be established when the pressure fluctuation was less than 0.001 MPa/h. Real-time pressure data were recorded using a data acquisition system.

At each pressure point, the system is maintained for 30–60 min until adsorption equilibrium is reached, and the data are recorded. The obtained data are used to plot adsorption isotherms, which are analyzed using models such as Langmuir to evaluate the adsorption performance of the coal.

### 2.3. Experimental Characterization of Sample Pore and Chemical Structure

(1) Pore Structure

The low-temperature CO_2_ adsorption experiment (LP-CO_2_-GA) can measure the pore volume and pore size distribution of the samples. The LP-CO_2_-GA experiment was conducted at the Key Laboratory of Coalbed Methane Resources and Reservoir Formation Processes of the Ministry of Education, China University of Mining and Technology, using a Quantachrome Autosorb-iQ surface area analyzer. The testing was performed according to the standard “Determination of Specific Surface Area and Pore Size Distribution of Rocks by Static Adsorption Capacity Method” (SY/T 6154-2019, 2019) [26]. The pore size distribution characteristic parameters of the coal samples were obtained using the NLDFT calculation model based on the experimental results [24,27,28].

(2) Chemical Structure

Fourier-transform infrared spectroscopy (FTIR) spectra can be used to analyze the distribution of functional groups and aromatic structures in coal samples. FTIR characterization was performed using a Bio-Rad FTS165 Fourier-transform infrared spectrometer (Bio-Rad, Hercules, CA, USA), with samples pressed into pellets with KBr. The instrument parameters were set as follows: a resolution of 1 cm^−1^, a scanning range of 4000 cm^−1^ to 400 cm^−1^, and both the sample and the blank KBr pellet were processed with 16 scans.

### 2.4. Simulation Methods

#### 2.4.1. Construction of Coal Macromolecular and Supramolecular Models

**Figure 2:**
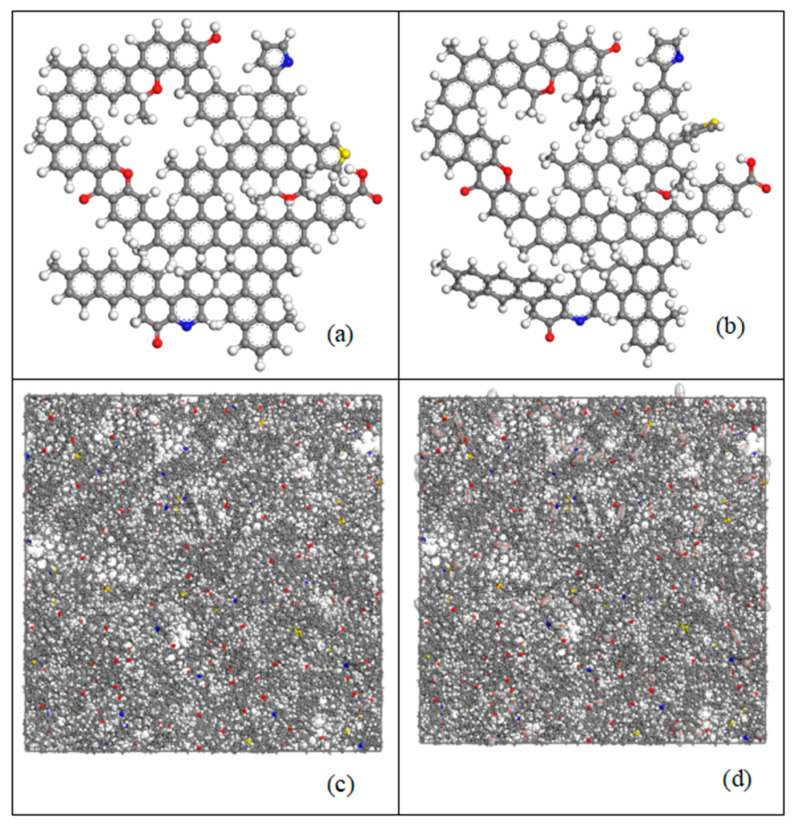
(**a**) Macromolecular structure of TL; (**b**) geometric optimization model of TL; (**c**) supramolecular structure of TL; and (**d**) ScCO_2_ injection model (C: gray; H: white; O: red; S: yellow; N: blue).

The macromolecular structure model is based on the TL coal structure model constructed by Bian et al. [29] (Figure 2a). The model construction and energy optimization were performed using the Forcite module in Materials Studio 2019 software. First, a Geometry Optimization task was used to optimize the geometry of the macromolecular structure. The COMPASS force field was selected, with the Q_e_ method used for charge equilibration, and the van der Waals and electrostatic interaction parameters set to Atom-based. Next, an Anneal task was performed on the optimized macromolecular structure. The initial temperature was set to 300 K, the intermediate temperature was set to 600 K, and the number of cycles was set to 10, with van der Waals and electrostatic interaction parameters also set to Atom-based [29]. The optimal geometric configuration of Tunlan coal was finally obtained (Figure 2b). Using the Construction task in the Amorphous Cell module, 30 coal macromolecules were introduced into a periodic box (5.25 nm × 5.26 nm × 5.26 nm) for processing. This resulted in the supramolecular structure model of TL coal (Figure 2c).

#### 2.4.2. Simulation of ScCO_2_ Injection Process

In the constructed supramolecular structure model, CO_2_ was injected using the Fixed Pressure module in Materials Studio software. The simulation was performed using an NPT ensemble, with the Andersen thermostat and Berendsen barostat for temperature and pressure control. Consistent with the experimental conditions, the simulation was conducted at a temperature of 50 °C and a pressure of 10 MPa. The simulation time was set to 250 ps, with a timestep of 1 fs and a sampling interval of 50 fs (Figure 2d) [30,31].

#### 2.4.3. Simulation Calculation of Pore Structure Parameters

The Poreblazer software (V4.0) was used to calculate the supramolecular structure model of TL coal before and after ScCO_2_ exposure. This provided pore structure parameters, which were used to analyze the changes in micropore volume, the micropore’s specific surface area, and the average micropore size of the coal supramolecular structure before and after ScCO_2_ exposure [32,33].

#### 2.4.4. ReaxFF-MD Simulation Calculations

After injecting CO_2_ into the supramolecular structure model, reactive force field molecular dynamics (ReaxFF-MD) dynamic calculations were used to determine bond orders and energy parameters between atoms in the supramolecular structure, as well as to simulate the formation and breaking of chemical bonds. This approach helps to describe the processes and mechanisms of chemical reactions.

In the ReaxFF-MD force field, the calculation formula for bond order functions is shown in Equation (1) [34].

(1) $${BO}_{ij}^{\prime }={BO}_{ij}^{\prime \sigma }+{BO}_{ij}^{\prime \pi }+{BO}_{ij}^{\prime \pi \pi }=exp[p_{bo1}{\left(\frac{r_{ij}}{r_{0}^{\sigma }}\right)}^{p_{bo2}}]+exp[p_{bo3}{\left(\frac{r_{ij}}{r_{0}^{\pi }}\right)}^{p_{bo4}}]+exp[p_{bo5}{\left(\frac{r_{ij}}{r_{0}^{\pi \pi }}\right)}^{p_{bo6}}]$$

Here, ${BO}_{ij}^{\prime \sigma }$, ${BO}_{ij}^{\prime \pi }$, and ${BO}_{ij}^{\prime \pi \pi }$ represent the bond orders for single, double, and triple bonds, respectively; $r_{0}^{\sigma }$, $r_{0}^{\pi }$, and $r_{0}^{\pi \pi }$ are the equilibrium distances for single, double, and triple bonds, respectively; and $p_{bo1}$–$p_{bo6}$ represents the empirical parameters regressed for the ReaxFF-MD force field.

In the ReaxFF-MD force field, the calculation formula for the energy function is shown in Equation (2) [35].

(2) $${\;E}_{system}=E_{bond}+E_{over}+E_{under}+E_{val}+E_{pen}+E_{tors}+E_{conj}+E_{vdWaals}+E_{Coulomb}$$

Here, $E_{bond}$ is the bond energy, $E_{over}$ is the over-coordination energy, $E_{under}$ is the under-coordination energy, $E_{val}$ is the bond angle energy, $E_{pen}$ is the penalty energy, $E_{tors\;}$ is the torsion energy, $E_{vdWaals}$ is the van der Waals energy, and $E_{Coulomb}$ is the Coulomb energy.

#### 2.4.5. Diffusion Coefficient Calculation Method

Different supramolecular structure models with varying reaction times were selected for CH_4_ adsorption simulations (using the same simulation method as described in Section 2.4.2). Subsequently, the mean square displacement (*MSD*) method was used to calculate the self-diffusion coefficient of CH_4_ [36,37].

The *MSD* can be calculated as follows [38]:

$$MSD\left(∆t\right)=\frac{1}{\tau -∆t}\int_{0}^{\tau -∆t}{\left[r(t-∆t)-r(t)\right]}^{2}dt={\langle \left[r(t-∆t)-r\left(t\right)\right]\rangle }^{2}$$

where τ is the total simulation time.

According to Einstein’s equation, the self-diffusion coefficient can be obtained [39,40].

$$D=\frac{1}{6}\underset{∆t\to \infty }{\lim}\frac{dMSD}{d∆t}$$

## 3. Results and Discussion

### 3.1. CO_2_ Adsorption Capacity of TL Coal

**Figure 3:**
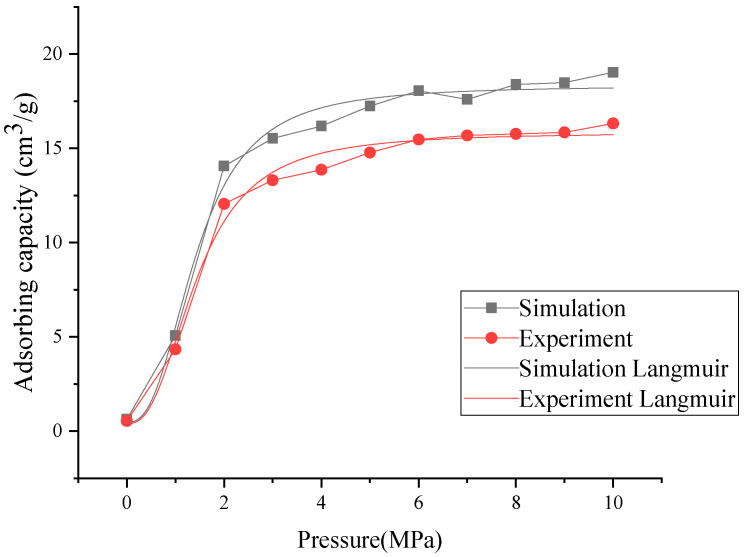
Relationship between the absolute adsorption capacity of TL coal and CO_2_ injection pressure.

The variation characteristics of the CO_2_ adsorption capacity and mechanical properties obtained by the experiment and molecular simulation have been compared in Figure 3. Bituminous coal exhibits a strong adsorption capacity for CO_2_, primarily due to its abundant micropores and mesopores, along with a high volatile matter content and carbon concentration. The pore structure of the coal provides a substantial surface area for CO_2_ molecules, with micropores playing a dominant role in the adsorption process. Compared to gases like CH_4_, CO_2_ molecules possess higher polarity and stronger intermolecular interactions (such as quadrupole moments), resulting in significantly higher adsorption capacities under the same pressure and temperature conditions.

Pressure notably influences the adsorption capacity: CO_2_ adsorption increases with pressure and approaches saturation at higher pressures. Experimental and simulation studies indicate that the CO_2_ adsorption isotherms of bituminous coal generally follow the Langmuir model, reflecting monolayer adsorption characteristics. Additionally, CO_2_ interacts readily with oxygen-containing functional groups in the coal (e.g., carboxyl and hydroxyl groups), enhancing adsorption stability.

Although the adsorption curves obtained by the experiment and simulation are analogous, the amount of CO_2_ adsorption obtained by simulation is greater than that obtained by the experiment. This is because the samples used in the experiment were powder coals, and the volume of pores in the experiment was smaller than that of the molecular simulation, resulting in an underestimation of CO_2_ adsorption capacity.

### 3.2. Changes in Pore Structure Characteristics of TL Coal

The pore structure of coal changes due to the swelling effect of ScCO_2_ and the mobilization effect on hydrocarbons. To analyze the impact of ScCO_2_ on micropores, mesopores, and macropores in the samples, the LP-CO_2_-GA experiment was used to test the pore volume and specific surface area parameters of TL, as shown in Table 2. After the ScCO_2_ treatment, the micropore volume of the sample increased from 0.021 to 0.025 cm^3^/g, an increase of 19.1%, and the micropore’s specific surface area increased from 58.93 to 65.52 m^2^/g, an increase of 11.2%. The mesopore volume increased from 2.29 × 10^−3^ to 2.65 × 10^−3^ cm^3^/g, an increase of 14.4%, with a corresponding increase in the mesopore’s specific surface area. The changes in macropore volume and specific surface area were not significant.

The pore structure parameters of the supramolecular model before and after ScCO_2_ treatment, calculated using Poreblazer software, are shown in Table 3. The molecular simulation data are generally consistent with the experimental results, indicating that the molecular simulation method accurately analyzes the pore size distribution in coal.

### 3.3. Changes in Chemical Characteristics of TL Coal

#### Characteristics of Chemical Structure Changes in Coal

The changes in aromatic structures and functional groups in TL coal were analyzed based on FTIR variation characteristics. The FTIR spectra of the coal samples mainly contain the absorbance peaks of aromatic structure (1600 cm^−1^), aromatic C-H groups (700–900 cm^−1^), oxygen-containing groups (1000–1800 cm^−1^), aliphatic hydrocarbon groups (2800–3000 cm^−1^), and hydroxyl groups (3000–3600 cm^−1^) [41,42]. The peak areas were calculated by integrating the absorbance over the specified wavenumber ranges, using a baseline correction to eliminate drift or noise. Peak area calculations were performed using Origin software (2018) which allows for the accurate quantification of functional groups. The coal structure is further divided as follows: the aromatic structure: (CH_2_)_n_ (n ≥ 4) (710–725 cm^−1^), 4H (735–750 cm^−1^), 3H (750–810 cm^−1^), 1H (860–900 cm^−1^), and 2H (810–860 cm^−1^); the oxygen-containing groups: alkyl ethers (1030–1040 cm^−1^), 2 °C-O secondary alcohols (1100–1125 cm^−1^), 2 °C-O phenols, ethers (1150–1250 cm^−1^), 2 °C-O in aryl ethers (1260–1351 cm^−1^), and small contents of carboxyl C=O (1650–1690 cm^−1^); the aliphatic hydrocarbons: (sym.) R_2_CH_2_ (2830–2900 cm^−1^) and (asym.) R_2_CH_2_/RCH_3_ (2910–2950 cm^−1^); and the hydroxyl groups: OH-N, ring hydroxyls, OH-O, OH-OH, OH-π, and -OH [43].

**Figure 4:**
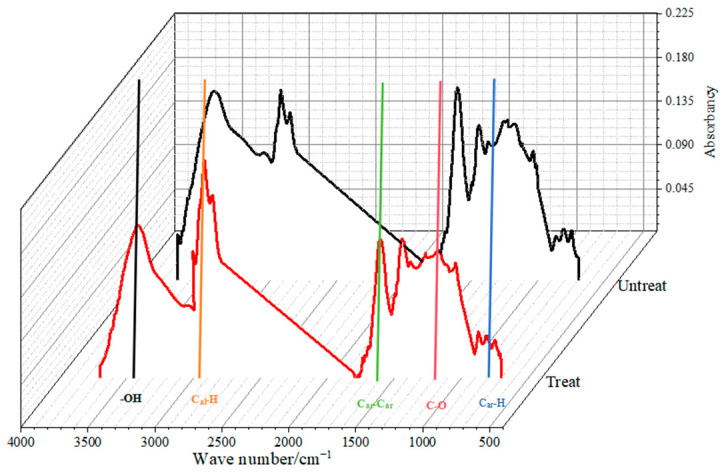
FTIR change characteristics before and after ScCO_2_ treatment.

After baseline correction of the FTIR spectra for the samples (Figure 4), the absorption bands corresponding to the C_ar_-C_ar_ skeletal vibrations of aromatic structures (1600 cm^−1^) and the C-H deformation vibrations of aromatic structures (900–700 cm^−1^) both weakened during the reaction, indicating a reduction in the aromatic structure content. The C-O absorption band of oxygen-containing functional groups (1800–1000 cm^−1^) also showed decreased intensity, suggesting a lower content of these functional groups. Conversely, the C_al_-C_al_ absorption band in the aliphatic structure (3000–2800 cm^−1^) exhibited increased intensity, indicating an increase in aliphatic structure content. The hydroxyl absorption band (3600–3000 cm^−1^) showed reduced intensity, suggesting a decrease in the content of hydroxyl functional groups.

Table 4 presents the changes in the characteristics of aromatic and various aliphatic structures extracted from the infrared spectra. The proportions of 4H and 3H in the aromatic structure decreased, while the proportions of (CH_2_)_n_, 2H, and 1H increased, indicating the degradation of the aromatic structure, where multi-ring aromatic structures may have opened to form benzene or bi-cyclic rings or fractured to become (CH_2_)_n_. In the oxygen-containing functional groups, the proportions of alkyl ethers and carboxyl groups decreased significantly, while the proportions of 2 °C-O secondary alcohols and 2 °C-O phenols increased. This may suggest that during the reaction between TL coal and CO_2_, the C=O and C-O-C bonds were broken, resulting in oxygen ions combining with H ions to form C-OH bonds. In the aliphatic structure, the proportion of RCH_3_ decreased while that of R_2_CH_2_ increased, likely due to the opening of the aromatic structure, leading to the formation of more R_2_CH_2_. Additionally, in the hydroxyl groups, the proportion of -OH increased while that of OH-π decreased, indicating a reduction in π bonds in the coal after exposure to ScCO_2_.

### 3.4. Change Mechanism of Micropore and Mesopore Structure in TL Coal

#### 3.4.1. Mechanism Analysis of Chemical Structure Change

**Figure 5:**
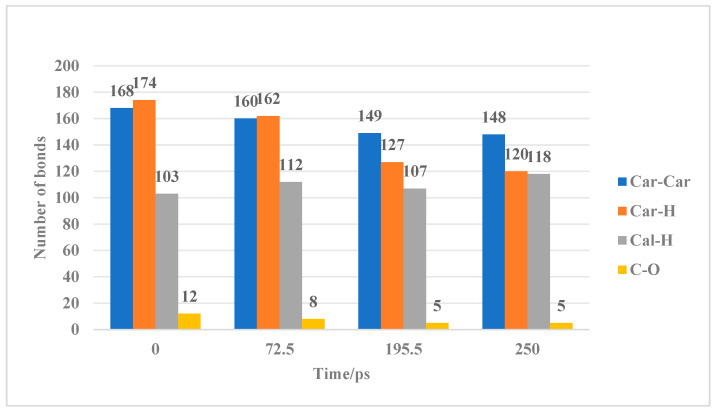
Characteristics of chemical bond changes in TL samples during ScCO_2_ treatment.

Due to factors such as baseline correction, peak overlap, and noise, the FTIR analysis method may struggle to provide precise quantitative results. Therefore, molecular simulation methods were combined to quantitatively analyze the changes in aromatic structures and functional groups in coal during the ScCO_2_ treatment, along with an examination of the mechanisms behind the chemical structure changes. Figures S1 and S2 show the variations in the supramolecular and macromolecular structures of TL coal after ScCO_2_ treatment. Figure 5 illustrates the changes in the number of different chemical bonds.

Based on the reaction characteristics of TL coal, the swelling reaction phase occurs from 0 to 72.5 ps, during which non-covalent bond breakage occurs between molecules, accompanied by the rupture of weak bridging bonds within the macromolecular network. The supramolecular structure of coal becomes more loosely entangled and expands outward. In the macromolecular structure, low-energy bonds at the edges break, including hydrogen bonds, π-π stacking interactions, and chemical bonds such as C_al_-O, C_al_-C_a_l, and C_a_l-H, while the molecular centroids remain unchanged. The C_ar_-C_ar_ bonds decreased from 168 to 160, C_ar_-H bonds decreased from 168 to 162, C_al_-H bonds increased from 103 to 112, and C-O bonds decreased from 12 to 8. From 72.5 to 250 ps, a mobilization effect of hydrocarbons primarily occurs. During the initial dissolution phase (72.5–195.5 ps), the segmental motion of the macromolecular structure in the coal is enhanced, facilitating the rearrangement of molecular segments. Due to the internal rotation of the σ bonds in the main chain, some segments of the molecule move relative to others while maintaining the center of mass, resulting in further outward expansion of the supramolecular structure of coal. This movement does not induce plastic deformation. The macromolecular structure of coal is primarily characterized by the breaking of linking bonds between molecules, which leads to the decomposition of the coal macromolecular structure into two sub-molecular fragments and the generation of additional small radical ions. These small radical ions collide with the aromatic structures in the coal, resulting in damage to those aromatic structures. The collisions between small molecules increase the system’s energy, leading to the outward expansion of the coal supramolecular structure until it reaches its maximum volume. The C_ar_-C_ar_ bonds decreased from 160 to 149, C_ar_-H bonds decreased from 162 to 147, Cal-H bonds decreased from 112 to 107, and C-O bonds decreased from 8 to 5. In the late dissolution phase (195.5–200 ps), segmental motion transitions to the movement of the entire macromolecular chain, which is known as coordinated segmental motion. The macromolecule gradually enters ScCO_2_ during this phase, where both decomposition and combination reactions occur. The impact of ScCO_2_ on the coal structure is most significant during the coordinated segmental motion; the supramolecular structure of coal no longer expands outward. The original functional groups and aromatic structures in coal are damaged, and the resulting free radical ions participate in addition or substitution reactions with the coal macromolecular structure, forming a small amount of new aromatic and aliphatic structures. The C_ar_-C_ar_ bonds decreased from 149 to 148, C_ar_-H bonds from 127 to 120, and C_al_-H bonds increased from 107 to 118, while the C-O bonds remained unchanged.

#### 3.4.2. Mechanism Analysis of Pore Structure Change

**Figure 6:**
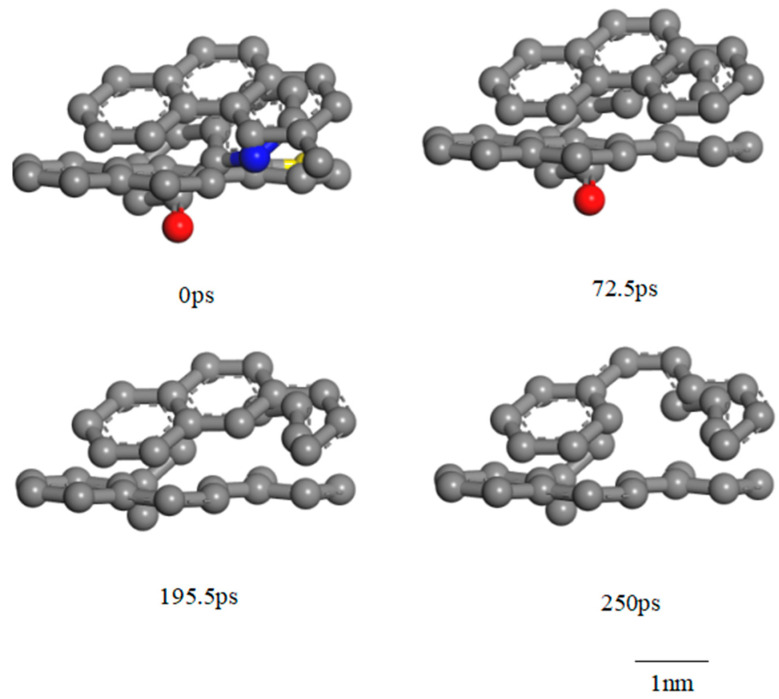
The process of new micropore formation during ScCO_2_ reactions (C: gray; O: red; N: blue; S: Yellow).

**Figure 7:**
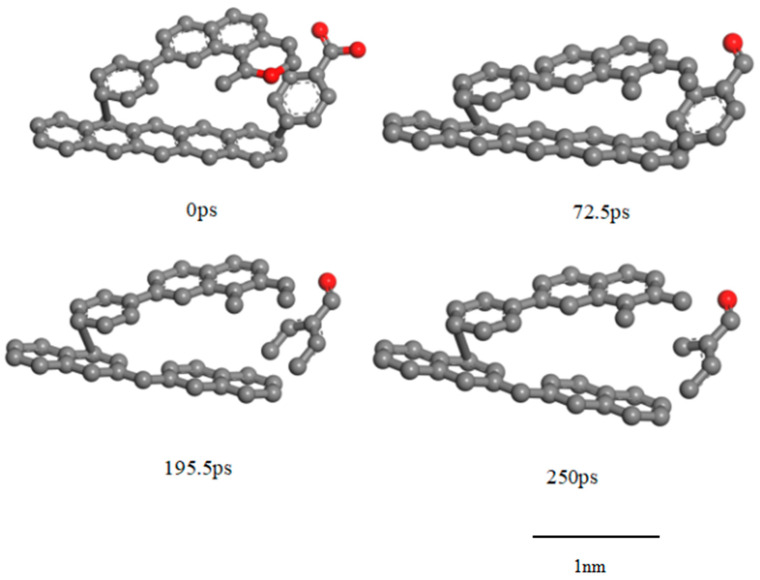
The process of micropore deformation during ScCO_2_ reactions (C: gray; O: red).

**Figure 8:**
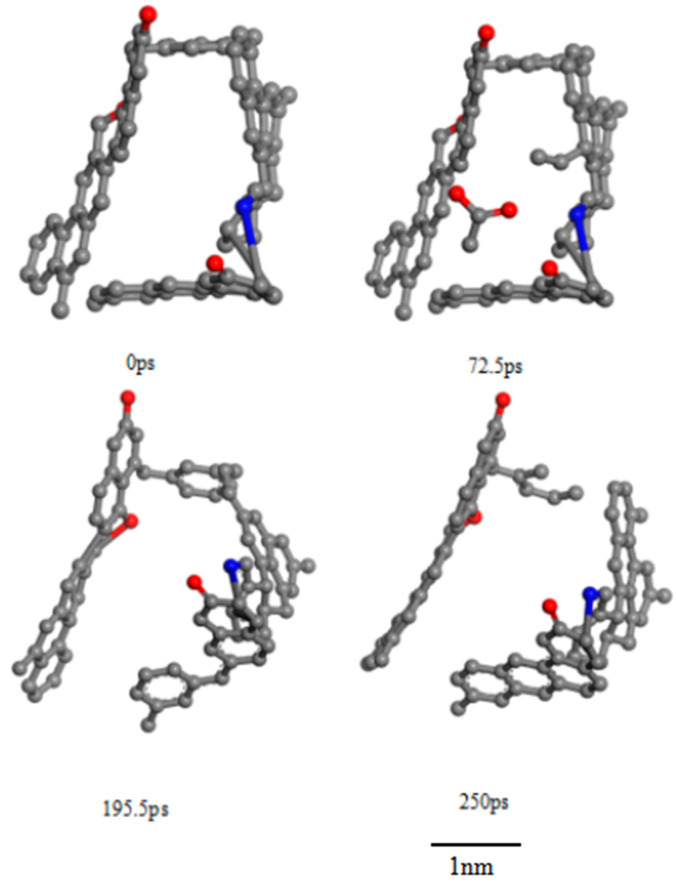
The process of mesopore deformation during ScCO_2_ reactions (C: gray; O: red; N: blue).

Figure 6, Figure 7 and Figure 8 show the characteristic changes in different pore sizes of TL during the reaction process analyzed by the ReaxFF-MD force field. In the initial stage of the reaction, a small number of new pores are generated, the micropore volume decreases, and the mesopore volume increases. In the mid-reaction stage, a large number of new pores are generated, the micropore volume increases, and the mesopore volume decreases. In the late reaction stage, no new pores are formed, the micropore volume remains relatively unchanged, and the mesopore volume increases. The chemical structure changes according to the micropores and mesopores during the reaction process.

Figure 6 illustrates the formation mechanism of micropores during the ScCO_2_ treatment process. The formation of micropores is primarily due to the breaking of low-energy chemical bonds, including the dehydrogenation of aliphatic structures and the destruction of unstable benzene rings and heteroatom-containing rings, which generates gaseous products and creates new pore structures. In the initial stage of the reaction, the solubility of ScCO_2_ in coal is relatively low, leading to the formation of only a few new pores. Although some low-energy chemical bonds (such as C-H and C-O) are broken and small molecules are released, the swelling of the coal and the increase in internal pore pressure cause the micropores that have already formed to be compressed, resulting in a decrease in micropore volume. At the same time, as the intermolecular distances in the coal increase, the volume of mesopores correspondingly increases. During the early dissolution phase, the solubility of ScCO_2_ significantly improves, allowing more ScCO_2_ molecules to interact with the coal’s chemical bonds, which in turn leads to the formation of a large number of new pores. In this phase, ScCO_2_ promotes the dehydrogenation of aliphatic structures, facilitating the removal of hydrogen atoms and the formation of unsaturated sites (free radical ions) while also releasing gaseous products. The release of these gases creates new voids that further increase the micropore volume. In addition, the destruction of the less stable benzene rings and heteroatom-containing rings results in the separation of molecular fragments, and the static release of gases from these fragments also contributes to the formation of additional micropores. In the late dissolution phase, the solubility of ScCO_2_ gradually decreases, leading to a slower reaction rate and fewer ScCO_2_ molecules participating in bond breakage. At this stage, the free radical ions generated in the early phases tend to collide, recombine, or undergo addition reactions to form new chemical structures rather than further breaking bonds, resulting in a significant reduction in the formation of new micropores and a relatively unchanged micropore volume. In summary, the evolution of micropores is characterized by a balance between the dissolution-driven breaking of low-energy bonds and the recombination of free radical ions and molecular reorganization. This process, which transitions from the initial formation of micropores to the abundant generation of micropores during the dissolution phase and finally to stabilization in the late stage due to diminishing solubility and radical recombination, effectively explains the gradual changes in the coal pore structure during the reaction with ScCO_2_ and provides a molecular-level understanding of the core drivers behind pore structure evolution.

Figure 7 illustrates the changes in micropores during the ScCO_2_ treatment process. In the early stages of the reaction, although the breaking of low-energy chemical bonds such as C-H and C-O occurs and small molecular gases (e.g., H_2_, CO, and CH_4_) are released, the overall expansion of the coal molecular structure and the increased internal pressure on the pore system play a dominant role, leading to the compression of existing micropores and a decrease in their diameter. This compression effect can be attributed to the swelling behavior of coal upon exposure to ScCO_2_, which increases intermolecular spacing and generates stress within the coal matrix. As a result, rather than immediately forming new pores, the existing micropores experience structural shrinkage due to the competition between pore expansion and coal matrix swelling. During the initial dissolution phase, as the solubility of ScCO_2_ in coal increases significantly, more ScCO_2_ molecules diffuse into the microporous structure and interact with the coal’s chemical bonds. This enhanced solubility promotes the dissolution of aliphatic and aromatic bonds within the micropores, leading to an increase in pore diameter. Specifically, the cleavage of aliphatic chains and weakly bonded aromatic rings facilitates the generation of unsaturated reactive sites, contributing to the release of small molecular byproducts. This process further weakens the structural integrity of the surrounding coal matrix, ultimately allowing micropores to expand. Additionally, as ScCO_2_ penetrates deeper into the coal matrix, it disrupts intermolecular interactions and reduces the effectiveness of van der Waals forces that hold the coal macromolecular framework together, thereby enhancing pore development. In the late dissolution phase, the influence of ScCO_2_ on micropores becomes more stabilized. The major structural changes during this stage occur within the aromatic framework, with only minor alterations observed in the micropore system. This is primarily because, at this stage, the remaining bonds available for breakage are those in the more stable aromatic structures, which require higher energy to cleave. Consequently, the reaction rate slows down, and the formation of new micropores is largely inhibited. Instead, previously generated free radicals and reactive sites undergo recombination or addition reactions, leading to structural rearrangements rather than continued pore development. The net result is a stabilization of the micropore system, with little additional variation in micropore volume or diameter.

Figure 8 illustrates the changes in mesopores during the ScCO_2_ treatment process, revealing a complex interplay between coal matrix swelling, chemical bond breakage, and structural rearrangements. In the early reaction stage, exposure to ScCO_2_ induces coal swelling, which increases intermolecular distances and leads to the expansion of mesopore volume. This phenomenon occurs because ScCO_2_ molecules diffuse into the coal structure and interact with macromolecular components, weakening interatomic interactions and causing lattice expansion. The increased spacing between molecular segments creates larger voids, thereby contributing to an initial increase in mesopore volume. During the initial dissolution phase, as ScCO_2_ solubility in coal increases significantly, it reacts with functional groups that contribute to mesopore formation. Specifically, low-energy bonds such as C-H and C-O, as well as weaker aliphatic linkages and oxygen-containing bridges, undergo cleavage. The disruption of these bonds leads to the release of small molecular gases such as CO, CH_4_, and H_2_, which can initially create new pore structures. However, at the same time, the breakdown of functional groups may result in pore blockages due to the collapse or partial closure of existing mesopores. This effect occurs when the fragmented molecular components reposition themselves within the pore structure, obstructing open pathways and leading to a temporary decrease in mesopore volume. In the late dissolution phase, structural rearrangements dominate, particularly within the aromatic framework. The displacement and repositioning of aromatic rings cause a weakening of the π–π stacking interactions and van der Waals forces that previously held these structures together. These interactions are critical in stabilizing the layered structure of coal macromolecules, and their weakening can result from geometric distortions, shifts in electron density, or changes in intermolecular forces induced by ScCO_2_ infiltration. As a result, the aromatic layers experience increased spacing, effectively enlarging the mesopore volume.

Compared with traditional experimental methods such as SEM, X-ray CT, and XRD, this study employs ReaxFF molecular dynamics simulations to investigate the interaction between ScCO_2_ and coal at the molecular scale, revealing the mechanisms of micropore and mesopore evolution. Experimental studies primarily analyze the structural changes in coal after ScCO_2_ exposure. For instance, Mohsen et al. observed increased surface smoothness in coal after CO_2_ saturation [44], Zhang et al. found that CO_2_-induced swelling generates numerous fractures in medium- and high-rank coals [45], and Major et al. reported that CO_2_ loosens the arrangement of aromatic microcrystals, leading to the expansion of micropores into mesopores [46]. However, these experimental techniques struggle to elucidate the effects of chemical bond cleavage, radical formation, and their roles in pore evolution. In contrast, ReaxFF simulations can precisely track how variations in ScCO_2_ solubility drive pore evolution at different stages, including initial micropore compression, significant micropore generation, and final stabilization. ReaxFF not only quantifies the bond dissociation energies of low-energy bonds such as C-H and C-O but also reveals the formation, recombination, and impact of radicals on pore expansion. Moreover, the simulation results indicate that ScCO_2_-induced aromatic structural reorganization and changes in π–π stacking interactions directly influence mesopore evolution, which is challenging to observe through experiments alone. Therefore, compared to experimental studies, ReaxFF simulations offer significant advantages in resolution, time scale, and mechanistic interpretation, providing a more precise microscopic understanding of the effects of ScCO_2_ on coal pore structure.

### 3.5. Analysis of Gas Diffusion Characteristics During the Reaction Process

**Figure 9:**
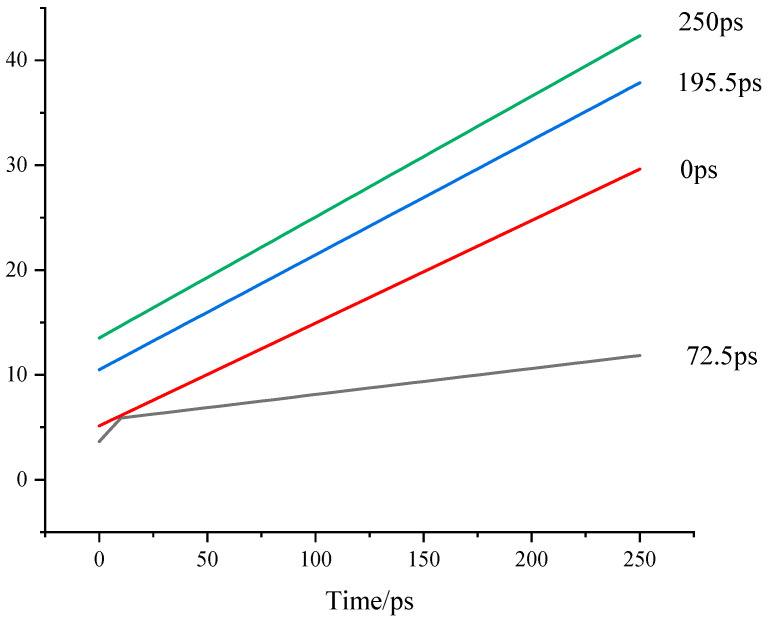
Changes in MSD of CH4 during ScCO_2_ reaction.

**Figure 10:**
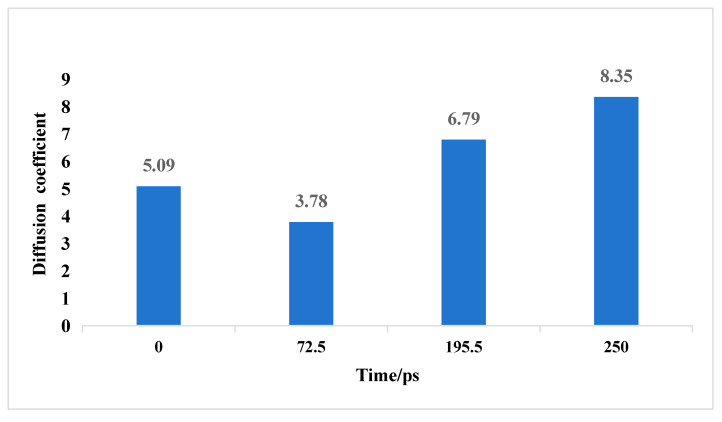
Self-diffusion coefficients change during the ScCO_2_ reaction.

This study investigated the effects of different reaction times on the pore size and chemical structure changes in TL coal and further explored how these changes influence the diffusion characteristics of gas (CH_4_) within TL coal at a fixed gas pressure of 5 MPa [18]. Analysis of the structural models after CH_4_ adsorption reveals significant changes in the pore size of coal with increasing reaction time, which in turn affects the diffusion behavior of gas molecules. Figure 9 and Figure 10 show the variations in the mean square displacement (MSD) and self-diffusion coefficient of CH_4_ at different reaction times.

It can be observed that both the MSD and self-diffusion coefficient of CH_4_ initially decrease and then increase with extended reaction time. This phenomenon indicates that during the interaction between ScCO_2_ and TL coal, the pore diameter of coal undergoes a process of first narrowing and then expanding. In the swelling phase, the coal sample experiences a swelling phase, where the breakage of low-energy bonds and the swelling compression effect lead to smaller pores, thereby reducing the diffusion capacity of gas within the coal. This reduction in pore size may be attributed to the rearrangement of the macromolecular structure during adsorption, resulting in further compression of existing intramolecular pores and hindering gas diffusion.

However, as the reaction time extends, hydrocarbon molecules within TL coal are mobilized by CO_2_, leading to the breakage of more chemical bonds and the emergence of larger pore sizes in the coal sample. At this stage, the original intramolecular pores evolve into intermolecular pores, which also contributes to an increase in the diameter of mesopores. These structural changes significantly enhance the self-diffusion coefficient of CH_4_, thereby improving the diffusion characteristics of gas molecules.

## 4. Conclusions

(1) After ScCO_2_ treatment, the micropore volume of the TL coal sample increased from 0.021 to 0.025 cm^3^/g (19.1%), while the micropore’s specific surface area increased from 58.93 to 65.52 m^2^/g (11.2%). The mesopore volume increased by 14.4%, from 2.29 × 10^−3^ to 2.65 × 10^−3^ cm^3^/g, with the mesopore’s specific surface area increasing from 0.69 to 0.73 m^2^/g. Changes in macropore volume and specific surface area were not significant. These alterations indicate a more complex pore structure, which can significantly influence methane adsorption and desorption in coal seams.
(2) Following ScCO_2_ exposure, the content of aromatic structures, oxygen-containing functional groups, and hydroxyl groups in TL coal decreased, while aliphatic structures increased. Notably, within aromatic structures, the proportions of (CH_2_)_n_, 2H, and 1H increased. In oxygen-containing functional groups, the proportions of 2 °C-O secondary alcohols and 2 °C-O phenols increased. In hydroxyl groups, the proportion of -OH increased, while within aliphatic structures, RCH_3_ decreased and R_2_CH_2_ increased.
(3) The evolution of coal pore structure under ScCO_2_ treatment progresses through distinct stages: Swelling Phase: The breakage of low-energy bonds reduces intramolecular spacing while increasing intermolecular spacing, forming new micropores. This leads to a reduction in the volume of existing micropores while increasing mesopore volume. Early Dissolution Phase: Further chemical bond breakage leads to increased micropore volume. Released small molecules occupy mesopore spaces, converting some mesopores into micropores. Later Dissolution Phase: Minimal chemical bond changes occur, but weakened π-π interactions and van der Waals forces between aromatic layers result in a further increase in mesopore volume. These findings provide a new perspective on the long-term impact of ScCO_2_ on coal matrix porosity, which is critical for assessing CO_2_ storage stability and gas migration behavior.
(4) During ScCO_2_ treatment, the mean square displacement (MSD) and self-diffusion coefficient of CH_4_ initially decrease and then increase. In the swelling phase, coal matrix expansion reduces pore volume, suppressing gas diffusion capacity. However, over time, the mobilization effect of CO_2_ leads to further pore expansion, ultimately enhancing the self-diffusion coefficient of CH_4_. This suggests that ScCO_2_ injection may initially hinder methane desorption but can improve overall gas recovery efficiency over prolonged exposure, providing insights into CO_2_-ECBM field applications.

## Figures and Tables

**Table 1:** Proximate analysis and ultimate analysis of sample.

Sample	Proximate Analysis (%)			Ultimate Analysis (%)					*R*_o,max_ (%)
	*M*_ad_	*A*_a__d_	*V*_daf_	*C*_daf_	*H*_daf_	*O*_daf_	*N*_daf_	*S*_daf_	
TL	0.38	6.91	23.9	86.34	3.96	6.14	1.09	4.47	1.45

*M*_ad_, moisture on an air-dried basis; *A_a_*_d_, ash on an air-dried basis; *V*_daf_, volatile matter on a dry and ash-free basis; wt% and wdaf%, weight percentage of various elements on a dry and ash-free basis. daf: dry, ash-free basis—a coal analysis basis that excludes moisture and ash content, reflecting the organic portion of the coal. ad: air-dried basis—a coal analysis basis that accounts for the coal’s natural moisture content after being air-dried.

**Table 2:** TL micropore volume and surface area parameters before and after the ScCO_2_ treatment experiment.

	*SSA*_mic_	*PV*_mic_	*SSA*_mes_	*PV*_mes_	*SSA*_mac_	*PV*_mac_
Untreated	58.93	0.021	0.69	2.29	0.004	0.001
Treated	65.52	0.025	0.73	2.65	0.004	0.001

*SSA*_mic_ = specific surface area of micropore (m^3^/g); *PV*_mic_ = pore volume of micropore (cm^3^/g); *SSA*_mes_ = specific surface area of mesopore (m^3^/g); *PV*_mes_ = pore volume of mesopore (10^−3^·cm^3^/g); *SSA*_mac_ = specific surface area of macropore (m^3^/g); *PV*_mac_ = pore volume of macropore (10^−3^·cm^3^/g).

**Table 3:** TL micropore volume and surface area parameters before and after the ScCO_2_ treatment simulation.

	*SSA*_mic_	*PV*_mic_	*SSA*_mes_	*PV*_mes_	*SSA*_mac_	*PV*_mac_
Untreated	63.56	0.025	0.82	2.48	0.005	0.001
Treated	69.47	0.031	0.92	2.87	0.005	0.001

**Table 4:** The changes in the characteristics of aromatic structure and various functional groups.

		Untreated		Treated	
	Assignment	Position, cm^−1^	Area Percentage, %	Position, cm^−1^	Area Percentage, %
Aromatic structure	(CH_2_)n	718.2	3.45	721.4	7.45
	4H	747.6	36.57	750.3	24.97
	3H	793.4	21.29	795.2	15.29
	2H	836.3	18.56	840.9	19.84
	1H	869.2	20.13	872.2	32.45
Oxygen-containing groups	alkyl ethers	1035.4	49.60%	1037.2	27.23%
	2 °C-O secondary alcohols	1119.8	6.26%	1123.5	24.54%
	2 °C-O phenols	1213.5	2.43%	1211.9	20.89%
	2 °C-O in aryl ethers	1298.2	1.65%	1292.4	2.63%
	carboxyl C=O	1683.7	40.06%	1689.2	22.71
Aliphatic hydrocarbons	(sym.) R_2_CH_2_	2842.5	18.45	2856.3	18.90
	(asym.) R_2_CH_2_	2925.4	36.73	2939.6	47.12
	(asym.) RCH_3_	2948.9	44.82	2950.1	33.98
Hydroxyl groups	-OH	3367.3	41.25	3376.2	59.19
	OH-π	3542.5	58.75	3556.6	40.81

## Data Availability

The original contributions presented in this study are included in the article/Supplementary Material. Further inquiries can be directed to the corresponding author.

## Supplementary Materials

The following supporting information can be downloaded at https://www.mdpi.com/article/10.3390/molecules30061200/s1: Figure S1. The TL coal supermolecular structure reaction process with ScCO_2_. Figure S2. The TL coal macromolecular structure reaction process with ScCO_2_.
