# Supplementary material for: Impact of Supercritical Carbon Dioxide on Pore Structure and Gas Transport in Bituminous Coal: An Integrated Experiment and Simulation
Source: Molecules. 2025 Mar 7;30(6):1200. doi: 10.3390/molecules30061200 (PMC11944282; doi:10.3390/molecules30061200)
Supplement: Supplementary file 1 [file molecules-30-01200-s001.zip › molecules-3489659-supplementary.pdf]

Supplementary Materials

Impact of Supercritical Carbon Dioxide on Pore Structure and Gas Transport in Bituminous Coal: An Integrated Experiment and Simulation

Kui Dong [1], Zhiyu Niu [1], Shaoqi Kong [2,*] and Bingyi Jia [3,4]

[1] College of Geological and Surveying Engineering, Taiyuan University of Technology, Taiyuan 030024, China; dongkui@tyut.edu.cn (K.D.); 15525037025@163.com (Z.N.)
[2] College of Mining Engineering, Taiyuan University of Technology, Taiyuan 030024, China
[3] School of Safety Science and Engineering, Xi’an University of Science and Technology, Xi’an 710000, China; jiabingyiccteg@126.com
[4] Xi’an Research Institute of China Coal Technology and Engineering Group Corp., Xi’an 710000, China
* Correspondence: kongshaoqi@tyut.edu.cn; Tel.: +86-136-6361-1856

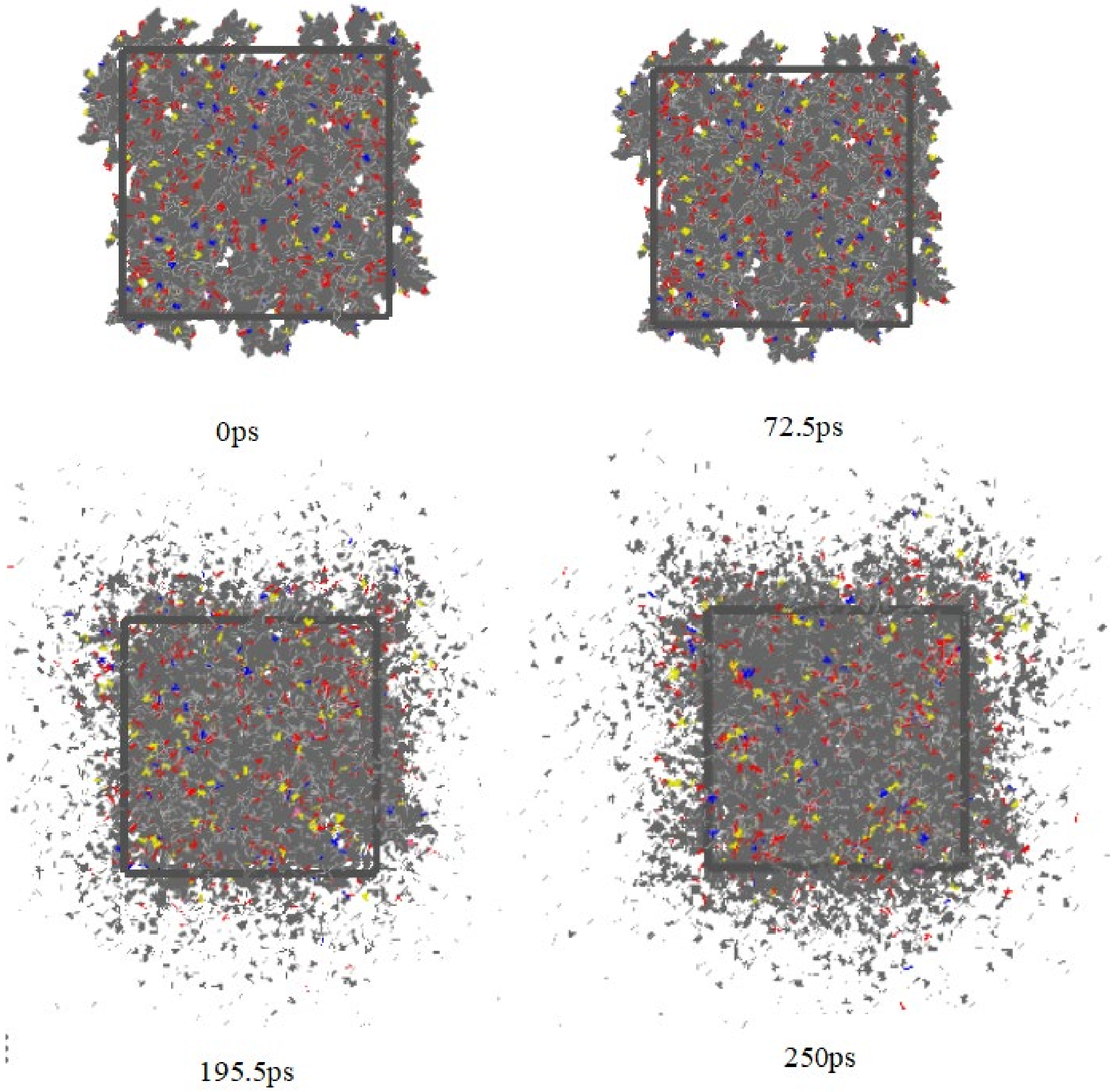

Figure S1. The TL coal supermolecular structure reaction process with ScCO$_2$(C: gray; H: white; O: red; S: yellow; N: blue)

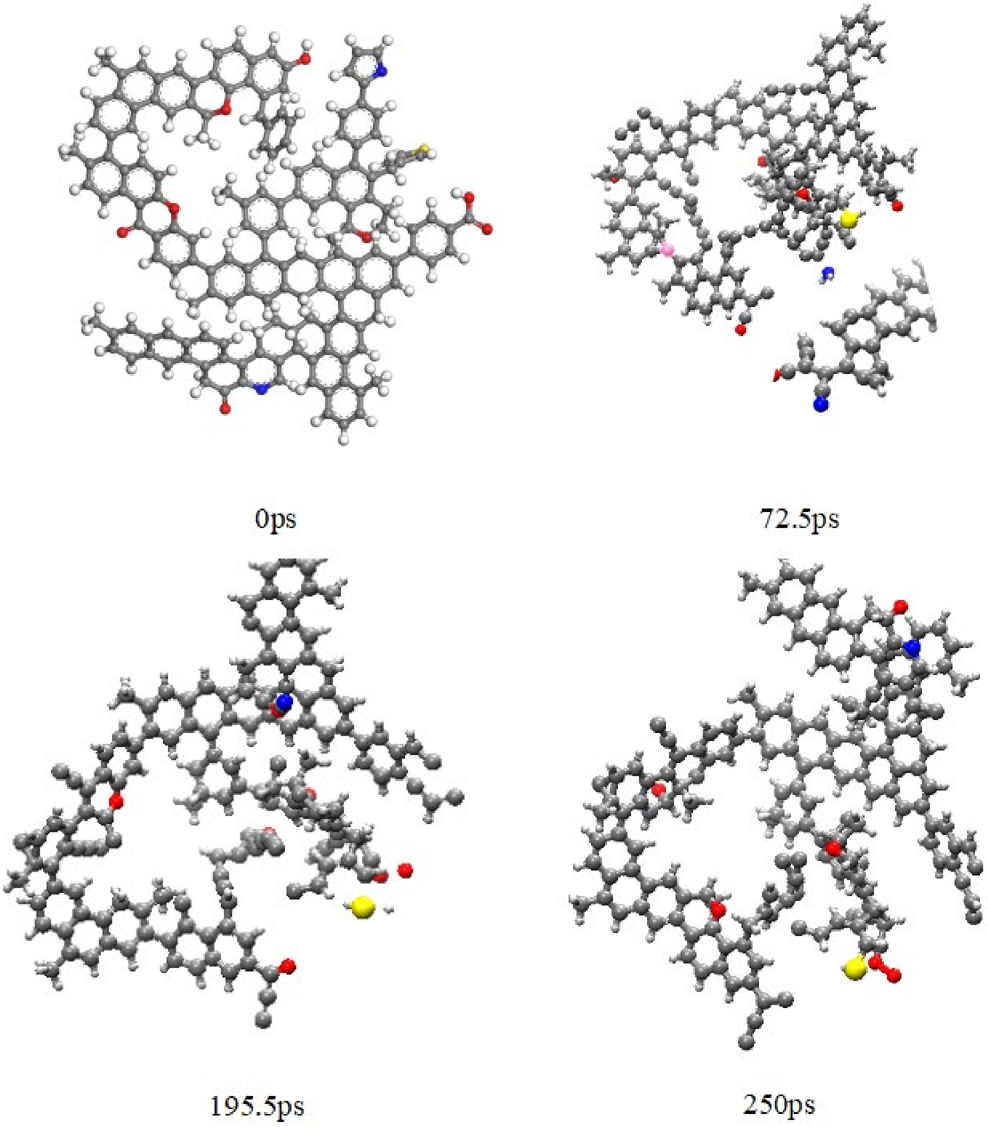

Figure S2. The TL coal macromolecular structure reaction process with ScCO$_2$(C: gray; H: white; O: red; S: yellow; N: blue)
